# Cross-Sectional Study of Clients’ Satisfaction With Outpatient and Inpatient Services of Public Health Facilities of a North Indian State

**DOI:** 10.1177/1178632920929969

**Published:** 2020-06-12

**Authors:** Manmeet Kaur, Abu Bashar, Tarundeep Singh, Rajesh Kumar

**Affiliations:** Department of Community Medicine and School of Public Health, Postgraduate Institute of Medical Education and Research, Chandigarh, India

**Keywords:** Client Satisfaction, public health services, medication side effects, staff behaviour, cleanliness

## Abstract

Satisfaction with health care services is a desired outcome of health care delivery. Nonetheless, there is scant information on client satisfaction with services provided in public health facilities in India. A cross-sectional study of persons attending public health facilities in Punjab, North India, was carried out in 2016. All district hospitals, subdistrict hospitals, 2 community health centres (CHCs), and 6 primary health centres (PHCs) were randomly selected from each of the 22 districts. A 60-item pre-tested and validated questionnaire was used to collect data. Participants (3278 outpatient department [OPD] and 1614 inpatient department [IPD]) visiting health care facilities were interviewed. Majority of OPD participants were satisfied with registration process, care providers, and personal issues like safety and security at the health facilities. Major domains of dissatisfaction were long waiting time and concern shown for patients during lab tests and x-rays. Most IPD participants were satisfied with care received from nurses and doctors, availability of medicines, and hospital environment. Domains of dissatisfaction were cleanliness of rooms and bathrooms and quietness at night. Varying levels of satisfaction were observed for experiences during stay, information about new medicine being given, pain control, and locomotion to bathroom or using bedpan. Around 71% were likely to recommend the health facility to others. Satisfaction with public health facilities is context dependent. Lack of drugs and supplies, poor information about medicines, long waiting time, poor cleanliness, lack of privacy, and peace were the major reasons for dissatisfaction in our study.

## Introduction

Patients’ or clients’ satisfaction with health care is an integral component of quality monitoring in health care systems:

Providers must get first-hand information from their clients, which should help them to reorient their services by adopting a more client centred approach, transforming their attitude and introducing a convivial ambience at health service outlets based on feedback of their clients.^1^

Donabedian defined patient satisfaction as patient-reported outcome measure while the structures and processes of care can be measured by patient-reported experiences.^2^ Moreover, quality assurance and accreditation process in most countries requires that satisfaction of clients be measured on a regular basis.^3^

Quality of health services has been traditionally based on professional practice standards. However, in recent times, patient’s perception about health care has been increasingly accepted as an important measure of quality of health care and a critical component of performance improvement and clinical effectiveness.^4^ Donabedian emphasised that client satisfaction is of fundamental importance as a measure of quality of care because it gives information on provider’s success at meeting those client values and expectations, on which client is the ultimate authority.^5,6^

Patients’ evaluation of care is a realistic tool to provide opportunity for improvement of care, enhancing strategic decision making, reducing cost, meeting patients’ expectations, framing strategies for effective management, monitoring performance of health plans, and provide benchmarking across health care institutions.^2,7-9^ In addition, patient satisfaction reflects patients’ involvement in decision making and their role as partners in improving the quality of health care services.^7,10^ There is a significant correlation between measuring patient satisfaction and patients’ compliance to treatment and continuity with health care providers.^11-13^

Majority of existing patient satisfaction studies have assessed overall satisfaction levels and paid little attention to satisfaction with specific domains of health care delivery. Domains of satisfaction have been viewed as multidimensional, which includes hospital structure, medical processes, and outcome of health care services.^14^ In recent years, the World Bank and other donors have been advising developing countries to ensure that limited resources not only have an optimal impact on population’s health at affordable cost but also that health services are client-oriented.^15-18^

Most research done on patient satisfaction with health services in India has been confined to family planning services. In this background, this study was conducted to measure the clients’ satisfaction with outpatient and inpatient services of public health facilities in a north Indian state.

## Material and Methods

The study was conducted in North Indian state of Punjab, situated between 29″30′N to 32″32′N latitude and 73″55′E to 76″50′E longitude with a total population of 28 million (Census 2011). Information was collected from users or clients seeking health care from public institutions: district hospitals (DHs), subdistrict hospitals (SDH), community health centres (CHCs), and primary health centres (PHCs). At each selected hospital/health facility, exit interviews were carried out using a structured study tool/interview schedule from persons seeking outpatient and inpatient health care. Each of the districts usually has one DH and one SDH, which represent secondary care institutions, and multiple CHCs and PHCs, which represent primary care institutions. Hence, all DHs and SDHs were included in the sample and 2 CHCs and 6 PHCs were randomly selected from the list of CHCs and PHCs in each of the 22 districts. The study was conducted between September 2015 and August 2016.

Sickness rate in Indian population at any given time averages around 10%. Assuming that these 10% population seeks outpatient department (OPD) health care, with power of 80% and precision of 5%, and 10% non-response rate, a sample size of 150 was required for each district. Hospitalisation rates in a population vary between 1% and 5% of the population. Assuming 5% hospitalisation, a sample size of 74 individuals from inpatient was arrived for each district. Hence, the total sample for each district was 150 + 74 = 224, and a sample of 224 × 22 = 4928 was required at the state level (Table 1).

**Table 1.** Sampling framework for the clients’ satisfaction survey in Punjab.

Type of facility	Number of patients from OPD	Number of patients from IPD
DH	30	20
SDH	30	20
2 CHCs	2 × 15 = 30	2 × 5 = 10
6 PHCs	6 × 10 = 60	6 × 4 = 24
Total	150	74
Grand total (for each district)	224	

Abbreviations: CHCs, community health centres; DH, district hospitals; IPD, inpatient department; OPD, outpatient department; PHCs, primary health centres; SDH, subdistrict hospitals.

From each of the institutions and health facilities, persons seeking care were selected through simple random sampling technique so as to represent the surgical and nonsurgical departments including emergency. In case of paediatric patients, adult caretakers were interviewed regarding their experiences in the hospital. Those clients who were mentally challenged/under influence of any drug or alcohol, or hearing impaired or unable to communicate due to any other reasons were excluded.

### Development of clients’ satisfaction tool

Four focussed group discussions (FGDs) were held in different settings of the state: 2 rural and 2 urban, one involving high-income group and other involving low-income group each to identify common issues in the health care institutions which determine or affect satisfaction with the services.

Existing scales were reviewed and adapted. Additional items were added to address issues identified during FGDs. A panel of experts (academicians, researchers, and health care providers) was then constituted to identify appropriate items to formulate the tool. The finalised tool for OPD participants had a 5-point Likert-type scale where questions and responses were categorised as excellent, good, fair, poor, and very poor. These 5 points were assigned scores from least favourable to most favourable (1-5). Each sub-component of the scale addressed different aspects of care giving, for example, experiences in the registration area, experience with nurses, experience and satisfaction with doctors, availability of medicines and infrastructure, and surrounding environment of the health facility. Similarly, for inpatient department (IPD) participants, a 4-point (always, usually, sometimes, and never) Likert-type scale questions were included in the scale. Five dimensions of perceived quality were identified – medicine availability, medical information, staff behaviour, doctor behaviour, and hospital infrastructure and relevant sections in tool were devoted to capture information on each of them.

The tool was developed in English with back-and-forth translated to Punjabi, the local language. The developed questionnaire was pilot tested in health facilities in neighbouring territory of Chandigarh for validity and reliability. Minor adjustments were made based on the pilot testing to ensure that all relevant domains had been covered and the language and format of questions were unambiguous.

### Data management and analysis

Data were analysed using Statistical Package for Social Sciences (SPSS) version 14.1. Descriptive analysis was employed to determine patients’ level of satisfaction. Level of satisfaction was categorised into 5 categories for OPD participants and 4 categories for IPD participants and percentage scores of each category were calculated.

### Ethical considerations

Written consent was obtained after intended participants were informed about the purpose and procedures of study. Privacy and confidentiality of data was ensured by masking personal identifiers like name and address and assigning a unique ID to each respondent. The questionnaires were digitised and field investigators filled in the answers as given by the participants online on tablet computers/mobile phones. This also ensured that the data recorded by investigators could not be accessed by anyone except authorised individuals. The study protocol was approved by the institute ethics committee (IEC) of Postgraduate Institute of Medical Education & Research, Chandigarh vide letter no PGI/IEC/2015/1129 dated July 21, 2015, Project no. P-229.

## Results

A total of 3278 patients or their caregivers from OPD and 1614 from IPD were interviewed for the study. Majority of patients belonged to rural areas (58.7% of IPD and 58.3% of OPD participants) and were females (57.4% for IPD and 50.1% for OPD). Different occupational groups seemed to use public health facilities in the similar proportions for IPD and OPD except professionals who used IPD more often than OPD. Younger age groups appeared to use IPD more often whereas the older age groups appeared to use OPD more often (Table 2).

**Table 2.** Sociodemographic characteristics of the study participants (N = 4928).

Variables	Sociodemographic characteristics	IPD (N = 1614) N (%)	OPD (N = 3278) N (%)
Place of residence	Rural	947 (58.7)	1911 (58.3)
	Urban	667 (41.3)	1367 (41.7)
Age (y)	18-30	632 (39.2)	143 (4.4)
	31-40	454 (28.1)	893 (27.2)
	41-50	244 (15.2)	880 (26.8)
	51-60	188 (11.6)	517 (15.8)
	Above 60	96 (5.9)	513 (15.6)
Gender	Female	924 (57.2)	1644 (50.1)
	Male	690 (42.8)	1634 (49.9)
Marital status	Married	1412 (87.5)	2557 (78.0)
	Single	202 (12.5)	721 (22.0)
Educational level	Below primary	381 (23.6)	846 (25.8)
	Up to middle	254 (15.7)	501 (15.3)
	Secondary	358 (22.2)	648 (19.8)
	Higher secondary	407 (25.2)	751 (22.9)
	Diploma/graduation and above	214 (13.3)	532 (16.2)
Occupation	Professional	286 (17.7)	235 (7.2)
	Farmer	156 (9.7)	428 (13.1)
	Skilled worker	258 (16.0)	470 (14.3)
	House wife	731 (45.3)	1159 (35.4)
	Retired	24 (1.5)	56 (1.7)
	Business person	153 (9.5)	400 (12.2)
	Unemployed	0 (0.0)	154 (4.7)
	Student	6 (0.3)	376 (11.5)
Religion	Hindu	624 (38.7)	1247 (38.0)
	Sikh	944 (58.5)	1906 (58.1)
	Others	46 (2.8)	125 (3.8)
Caste/category^a^	General	641 (39.7)	1423 (43.4)
	Other backward castes	313 (19.3)	744 (22.7)
	Scheduled castes	638 (39.5)	1068 (32.6)
	Not applicable	22 (1.5)	38 (1.5)
Type of house	Kaccha house	158 (9.8)	178 (5.4)
	Kaccha-pacca house/pacca house	1456 (90.2)	3100 (94.6)
BPL Card	Yes	181 (11.2)	346 (11)
	No	1433 (88.8)	2932 (89)
Monthly household income (in INR)	0-7000	400 (24.8)	702 (21.4)
	7001-15 000	902 (55.9)	1674 (51.1)
	15 001-30 000	282 (17.5)	784 (23.9)
	30 001-60 000	30 (1.8)	118 (3.6)

Abbreviations: BPL, below poverty line; INR, Indian National Rupees; IPD, inpatient department; OPD, outpatient department.

a As per the categories defined by central and state governments.

### Outpatient department

Participants had mean age of 34.6 ± 12.6 years, majority had education above secondary level (60%), were married (87%), housewives (35.4%), and above poverty line (89%). Around 75% of the participants had monthly household income above 7000 INR (Table 2).

Less than 5% of the OPD participants rated speed of registration as either poor or very poor. Similarly, very few were dissatisfied with courtesy of the staff in the registration area with less than 3% rating them as either poor or very poor. Majority were also satisfied with other domains of registration like comfort of the waiting area, waiting time before going to check-up room, comfort and pleasantness of the check-up room, friendliness/courtesy of the nurse/assistant, concern the nurse/assistant showed for the problem, and waiting time in the exam room before being seen by the care provider (Table 3).

**Table 3.** Rating of different aspects of registration process by OPD participants.

	Variables	Excellent N (%)	Good N (%)	Fair N (%)	Poor N (%)	Very poor N (%)
1.	Speed of registration	904 (27.6)	1305 (39.8)	909 (27.7)	144 (4.4)	16 (0.5)
2.	Courtesy of staff in the registration area	958 (29.3)	1336 (40.7)	883 (26.9)	97 (2.9)	4 (0.1)
3.	Comfort and pleasantness of the waiting area	913 (27.8)	1084 (33.1)	1069 (32.6)	200 (6.1)	12 (0.4)
4.	Length of wait before going to check-up room	890 (27.2)	1249 (38.1)	893 (27.2)	227 (6.9)	19 (0.6)
5.	Comfort and pleasantness of the check-up room	953 (29.1)	1174 (35.8)	963 (29.4)	179 (5.5)	9 (0.3)
6.	Friendliness/courtesy of the nurse/assistant	1050 (32.0)	1470 (44.8)	664 (20.2)	89 (2.8)	5 (0.2)
7.	Concern the nurse assistant showed for your problem	1043 (31.8)	1149 (35.1)	947 (28.9)	132(4.0)	7 (0.2)
8.	Waiting time in the exam room before being seen by the care provider	951 (29.0)	1162 (35.4)	1000 (30.5)	149 (4.5)	16 (0.6)

Abbreviation: OPD, outpatient department.

**Figure 1.**
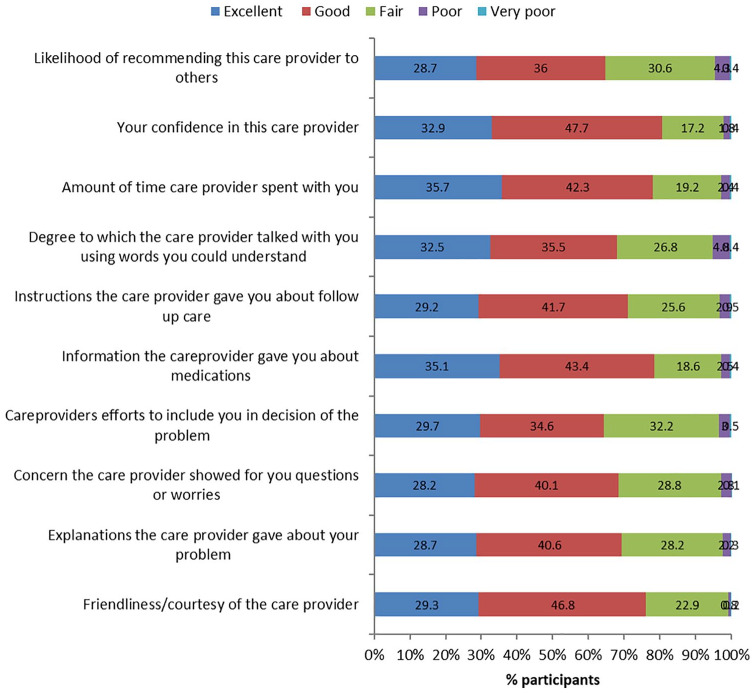
Rating of the various domains of care received from the care provider by the OPD participants. OPD indicates outpatient department.

Majority of the participants were satisfied with various aspects of care provision like friendliness/courtesy of the care provider, explanations the care provider gave about the problem, concern the care provider showed for the questions or worries, care provider’s efforts to include the patient in decisions about the treatment, information the care provider gave about medications and follow-up care, and so on (Figure 1).

Overall, majority (80%) were satisfied with the care received from the care providers with less than 3% being either completely or somewhat unsatisfied.

**Figure 2.**
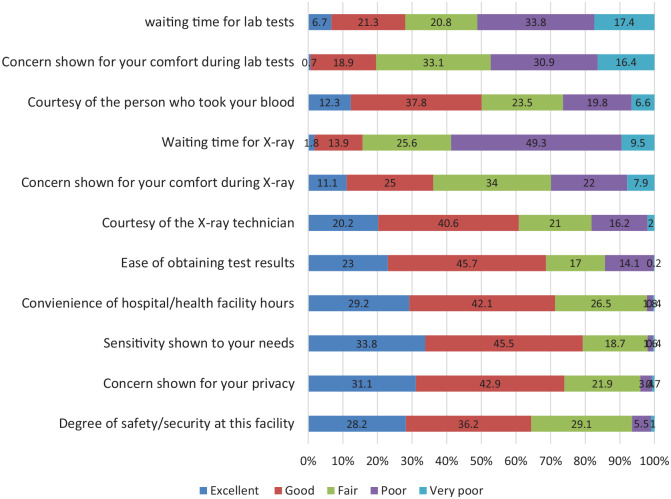
Rating of various domains of personal issues and laboratory/radiology services by the OPD participants. OPD indicates outpatient department.

Majority of the participants were satisfied with the various personal issues like convenience of hospital/health facility hours, sensitivity shown to the needs and concern shown for privacy. However, around 7% of the participants rated the degree of safety/security at the hospital/health facility as poor or very poor (Figure 2).

A total of 693 (21.1%) out of the 3278 OPD participants availed laboratory/radiology services from the concerned health facilities. Major domains of dissatisfaction were waiting time for lab tests (51.2% rating it as either poor or very poor), waiting time for x-rays (58.8% rating it as either poor or very poor), concern shown for patient’s comfort during the lab tests (47.3% rating it as either poor or very poor), and concern shown for patient’s comfort during x-ray (31.9% rating it as either poor or very poor) (Figure 2). However, majority were satisfied with the dealing of staff during the lab/radiology tests with only around 7% only being unsatisfied.

**Figure 3.**
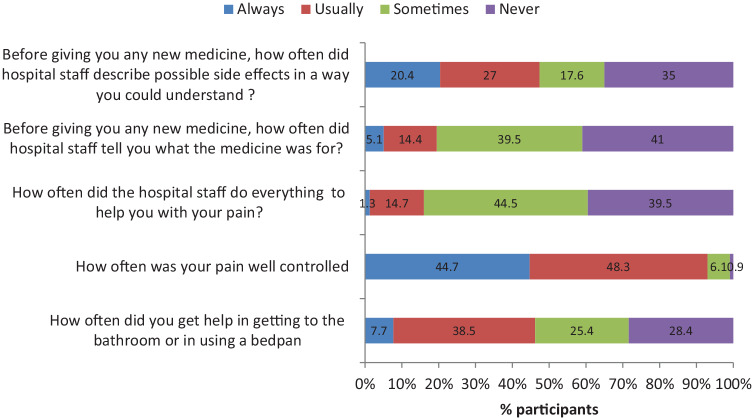
Various experiences of the IPD participants during hospital stay. IPD indicates inpatient department.

In the OPD, majority were satisfied with the politeness of the hospital staff as only 1.8% of the participants rated it as poor or very poor. Only 6% of the respondent rated overall cleanliness of the hospital/health facility as poor or very poor. Similarly, majority (72.2%) rated the overall care received during the visit as either good or very good and only 4.8% of the participants said that their likelihood of recommending the hospital to others was poor or very poor (Figure 3).

## Inpatient Department

Mean age of participants from IPD was 36.56 ± 21.23 years with majority being in age range of 31 to 50 years. Around 59% of participants had education above secondary level. About 11% of participants were below poverty line (BPL) and monthly household income was more than INR 7000 for approximately more than 75% of the participants (Table 2).

Majority (78.2%) of participants reported to be satisfied with overall care received from nurses with less than 3% being unsatisfied (Figure 3). The nurses treated the participants with courtesy, listened to them carefully, and explained the things in an understandable way and participants got help as soon as wanted from the nurses as less than 1% of total participants answered to these questions as never (Table 4).

**Table 4.** Rating of different domains of care received from nurses and doctors and of the hospital environment by the IPD participants.

	Variables	Always N (%)	Usually N (%)	Sometimes N (%)	Never N (%)
1.	How often did nurses treat you with courtesy and respect?	731 (45.3)	739 (45.8)	130 (9.9)	14 (0.9)
2.	How often did nurses listen carefully to you?	798 (49.4)	706 (43.7)	99 (6.0)	13 (0.9)
3.	How often did nurses explain things in a way you could understand?	836 (51.8)	626 (38.8)	138 (8.5)	14 (0.9)
4.	How often did you get help as soon as you wanted it from hospital staff?	829 (51.4)	632 (39.2)	142 (8.7)	11 (0.7)
5.	How often did doctors treat you with courtesy and respect?	801 (49.6)	728 (45.1)	80 (4.9)	5 (0.3)
6.	How often did doctors listen carefully to you?	889 (55.1)	667 (41.3)	53 (3.3)	5 (0.3)
7.	How often did doctors explain things in a way you could understand?	881 (54.6)	570 (35.3)	157 (9.7)	6 (0.4)
8.	How often your room and bathroom were kept clean?	650 (40.3)	761 (47.1)	168 (10.4)	35 (2.2)
9.	How often was the area around your room quiet at night?	939 (58.2)	567 (35.1)	97 (6.0)	10 (0.7)

Abbreviation: IPD, inpatient department.

Similarly, majority (80%) of the participants reported satisfaction with the overall care received from the doctors with less than 2% being unsatisfied. The doctors treated the participants with courtesy and respect, listened to them carefully, and explained things in an understandable way to the patients (Table 4).

Around 2% of participants reported that room and bathrooms were never clean and another 10% reported them to be clean sometimes only. Similarly, around 1% and 6% of the participants replied that area around their room was never or only sometimes quiet at night, respectively (Table 4). Overall, only 46% of the participants were satisfied with the hospital environment.

With domains of other experiences during hospital stay, out of 130 patients who required help going to bathroom or using a bedpan, 23.1% of the participants told that they never got help from nurses or other hospital staff and around 21% replied as getting the help sometimes only. Similarly, among the 658 patients requiring medicines for pain, around 1% and 6% of them reported to have never having pain well controlled and having pain controlled sometimes, respectively. Out of 729 participants who said they were given medicines which they had not taken before, around 5% and 14% of participants replied that they were never and sometimes only told what the medicine was for, respectively. Around 35% of the participants who were given any new medicines replied that they were not told about the possible side effects of the medicines in a way they could understand. However, 84% of the inpatient participants were satisfied with the availability of medicine in the hospital/health facility and around 71% of them were likely to recommend the hospital/health facility for admissions to others (Table 4)

## Discussion

Measurement of patients’ satisfaction is important for improving services and strategising goals for all health care organisations.^19^

In our study, majority (97%) of OPD participants were satisfied with overall care received. This is much higher than found in some of the other studies from developing world.^20-26^ Previous studies from India have reported patient satisfaction score ranging from 60% to 88%.^27-31^ Higher level of satisfaction among the OPD participants in our study may be attributable to availability of free medicines and low cost of laboratory tests. Politeness and courtesy are context and culture specific and cannot be directly compared across cultures. However, it is important that the clients perceive the behaviour of service providers as acceptable.

A vast majority (98%) of the participants in our study were satisfied with overall politeness of the hospital/health facility staff and around 95% of the OPD participants were likely to recommend the hospital/health facility to others. In a study to assess the level of satisfaction of patients attending a public tertiary hospital in Nigeria, 78.5% of the participants were satisfied with the hospital services and 91.7% were likely to recommend the health facility to a friend.^22^ In another study to assess degree of clients’ satisfaction among patients attending government health facilities in rural Bangladesh, only 68.9% of the participants expressed satisfaction with the provider’s usual behaviour.^23^

Around 7.5% of OPD participants were dissatisfied with long waiting time for consultation. In the study to evaluate patients’ satisfaction with quality of care provided at National Health Insurance Scheme (NHIS) clinic of a tertiary hospital in South-Eastern Nigeria, 48.3% of participants were dissatisfied with long waiting time.^24^ In a study from Bangladesh, 28.2% of users were not satisfied with the time they waited to receive care.^23^ In another study to assess client satisfaction with health services of a specialised tertiary care Centre in Ethiopia, dissatisfaction was reported to be highest (46.9%) by participants with waiting time.^25^

In our study, majority (98%) of OPD participants were satisfied with overall care received from care provider and majority (97%) were also satisfied with time the care providers spent with patients. In a study assessing satisfaction of patients with primary health care services in Saudi Arabia, 16.7% of satisfied and 38.9% of dissatisfied clients complained that physicians did not satisfactorily explain their health problems and treatments.^26^ In contrast, 96% of OPD participants in our study were satisfied with explanation the care provider gave to them about their problems and 95% could understand the words used by care providers.

In our study, majority (97.2%) of the OPD participants found the hospital/health facility hours convenient. Around 95% were satisfied with concern shown for their privacy. This is in contrast to a study from Bangladesh where a significant proportion of users (34.2%) were unsatisfied with the length of time the facilities were open to the public and only 45.1% of the clients were satisfied with the privacy maintained at the health facility.^23^

With inpatient services, 78% of participants reported to be satisfied with overall care received from nurses and 80% of participants reported to be either completely or somewhat satisfied with care received from the doctors. In the study by Rajkumari and Nula^32^ done in a tertiary care health facility from North East India and by Malangu and Westhuisen^33^ done in a DH of South Africa, 32.5% and 50% of the patients, respectively, were satisfied with the overall inpatient care which is lower than seen in our study. In another study by Mishra and Mishra^34^ conducted in a tertiary care private hospital of North India, the nursing services satisfied 80% of the participants while 92% were satisfied with explanation about disease and treatment by doctors. However, in the same study, behaviour of nurses, doctors, and orderlies satisfied 92%, 92%, and 83% of people, respectively.^34^ The somewhat higher level of satisfaction with the behaviour of nurses and doctors in the above study compared to ours might be due to differences in the study populations and the different study settings.

Another study done among patients admitted to obstetrics and gynaecology wards of public hospitals of Ethiopia reported overall satisfaction rate of 79.7% similar to our findings.^35^ However, concerning hospital staff informing to clients what the medicine was before giving new medicine, describing possible side effects of the medications in ways clients could understand, and cleanliness of toilet and washroom were the areas in which clients were dissatisfied in the study similar to our findings.^35^

With respect to hospital environment, around 46% of participants were satisfied with hospital environment, with 12% reported problem with cleanliness of toilets and rooms and 7% reported problem with quietness during night. Similar findings were reported by Mishra and Mishra^34^ in their study in which only 49% of the inpatients were satisfied with cleanliness of the toilets. However, in the study by Malangu and Westhuisen,^33^ 80% of participants were happy with cleanliness of wards, bedding and ablution facilities, as well as safety at night. The results of this study confirm that perception and judgement of quality are highly individualistic, dynamic and consequently client satisfaction has an important reflection on the quality of health care process.

## Conclusions and Recommendations

Majority of patients using outdoor and indoor services were satisfied with the care received and the behaviour of the received and behaviour of hospital staffs. Registration process needs to be streamlined to reduce waiting time and delays. In laboratory services, particularly the waiting time may be reduced and patients’ comfort during lab tests and x-ray may be improved for improving satisfaction of clients. Majority of participants, both OPD and IPD, agreed that behaviour of hospital staff was good, doctors and nurses spent adequate time with patients, and detailed information about illness and treatment was provided to them by doctors. Attention should be given to the perceived feeling of lack of personal security and cleanliness at health facilities. Before prescribing a new medicine to a patient, the possible side effects and purpose of giving the medicine should also be explained to them. Finally, a system for clients’ feedback may be institutionalised at all health facilities to improve quality of care.

## Supplemental Material

Questionnaire – Supplemental material for Cross-Sectional Study of Clients’ Satisfaction With Outpatient and Inpatient Services of Public Health Facilities of a North Indian State Click here for additional data file. Supplemental material, Questionnaire for Cross-Sectional Study of Clients’ Satisfaction With Outpatient and Inpatient Services of Public Health Facilities of a North Indian State by Manmeet Kaur, Abu Bashar, Tarundeep Singh and Rajesh Kumar in Health Services Insights
