# Supplementary material for: Cross-Sectional Study of Clients’ Satisfaction With Outpatient and Inpatient Services of Public Health Facilities of a North Indian State
Source: Health Serv Insights. 2020 Jun 12;13:1178632920929969. doi: 10.1177/1178632920929969 (PMC7294374; doi:10.1177/1178632920929969)
Supplement: Questionnaire – Supplemental material for Cross-Sectional Study of Clients’ Satisfaction With Outpatient and Inpatient Services of Public Health Facilities of a North Indian State [file Questionnaire.pdf]

1. Sociodemographic Characteristics		
1.	Age (in completed years)	
2.	Gender	1. Male 2. Female
3.	Religion	1. Hindu<br>2. Muslim<br>3. Christian<br>4. Sikh<br>5. Jain<br>6. Others
4.	Degree in Pharmacy	1. Graduate<br>2. Post graduate<br>3. Diploma<br>4. None
5.	Health facility currently working at	1. District hospital<br>2. CHC<br>3. PHC
6.	Work experience	1. < 6 months<br>2. 6-12 months<br>3. >12 months

2.Dispensing pattern of antibiotics		
1.	For which ailments are antibiotics prescribed the most?	
2.	Which antibiotics are prescribed the most?	
3.	Do you dispense medicines without prescription?	1. Yes<br>2. No
4.	If yes,<br>a) Do you dispense antibiotics without prescription?<br>b) For how many days it is prescribed?	1. Yes<br>2. No
5.	Do you explain the patient how medicine is to be taken?	1. Yes<br>2. No
6.	Do you provide the patients' necessary information regarding possible side effect of the drugs every time?	3. Yes<br>4. No
7.	Source of current drug knowledge	1. On the job experience<br>2. Medical representatives<br>3. Doctor<br>4. Books/social media
8.	Do you maintain a stock register?	

Information from patient's prescription

1.	Age (in completed years)	
2.	Gender	1. Male<br>2. Female
3.	Diagnosis/complaint	
4.	Were antibiotics prescribed?	1. Yes<br>2. No
5.	If yes, what antibiotic was prescribed?	
6.	Dose of antibiotic prescribed?	
7.	For how many days antbiotic is prescribed?	
8.	Medicine prescribed by whom?	1. Government practitioner<br>2. Private practitioner<br>3. Other
9.	Type of registration?	1. Old<br>2. New
